# Biologically informed NeuralODEs for genome-wide regulatory dynamics

**DOI:** 10.21203/rs.3.rs-2675584/v1

**Published:** 2023-03-14

**Authors:** Intekhab Hossain, Viola Fanfani, John Quackenbush, Rebekka Burkholz

**Affiliations:** 1 Department of Biostatistics, Harvard T.H. Chan School of Public Health, Boston, MA, USA.; 2 Helmholtz Center for Information Security (CISPA), Saarbrücken, Germany.

## Abstract

Models that are formulated as ordinary differential equations (ODEs) can accurately explain temporal gene expression patterns and promise to yield new insights into important cellular processes, disease progression, and intervention design. Learning such ODEs is challenging, since we want to predict the evolution of gene expression in a way that accurately encodes the causal gene-regulatory network (GRN) governing the dynamics and the nonlinear functional relationships between genes. Most widely used ODE estimation methods either impose too many parametric restrictions or are not guided by meaningful biological insights, both of which impedes scalability and/or explainability. To overcome these limitations, we developed PHOENIX, a modeling framework based on neural ordinary differential equations (NeuralODEs) and Hill-Langmuir kinetics, that can flexibly incorporate prior domain knowledge and biological constraints to promote sparse, biologically interpretable representations of ODEs. We test accuracy of PHOENIX in a series of *in silico* experiments benchmarking it against several currently used tools for ODE estimation. We also demonstrate PHOENIX’s flexibility by studying oscillating expression data from synchronized yeast cells and assess its scalability by modelling genome-scale breast cancer expression for samples ordered in pseudotime. Finally, we show how the combination of user-defined prior knowledge and functional forms from systems biology allows PHOENIX to encode key properties of the underlying GRN, and subsequently predict expression patterns in a biologically explainable way.

## Background

Biological systems are complex with phenotypic states, including those representing health and disease, defined by the expression states of the entire genome. Transitions between these states occur over time through the action of highly interconnected regulatory processes driven by transcription factors. Modeling molecular mechanisms that govern these transitions is essential if we are to understand the behavior of biological systems, and design interventions that can more effectively induce a specific phenotypic outcome. But this is challenging since we want not only to predict gene expression at unobserved time-points, but also to make these predictions in a way that explains any prior knowledge of transcription factor binding sites. Models that accurately encode such interactions between transcription factors and target genes within gene regulatory networks (GRN) can provide insights into important cellular processes, such as disease-progression and cell-fate decisions [1–4].

Given that many dynamical systems can be described using ordinary differential equations (ODEs), a logical approach to modeling GRNs is to estimate ODEs for gene expression using an appropriate statistical learning technique [3–6]. Although estimating gene regulatory ODEs ideally requires time-course data, obtaining such data in biological systems is difficult (if not impossible given the destructive nature of the associated assays). One can instead use pseudotime methods applied to cross-sectional data to order samples and subsequently estimate ODEs that captures the regulatory structure [7, 8].

While a variety of ODE estimation methods have been proposed, most suffer from key issues that limit their applicability in modeling genome-wise regulatory networks. Some systems biology models formulate ODEs based solely on biochemical principles of gene regulation, and use the available data to parameterize these equations [9]. However, such methods impose several restrictions on the ODEs and cannot flexibly adjust to situations where the underlying assumptions do not hold; this increases the risk of model misspecification and hinders scalability to large networks, particularly given the enormous number of parameters necessary to specify a genome-scale model [10, 11]. Other methods are based on non-parametric methods to learning regulatory ODEs, using tools such as sparse kernel regression [3], random forests [5], variational autoencoders [7, 12, 13], diffusion processes [4], and neural ordinary differential equations [6, 14], but these fail to include biologically relevant associations between regulatory elements and genes as constraints on the models.

These latter models can be broadly placed into two classes based on the inputs required to estimate the gradient f of the gene regulatory dynamics, where $f(x)=\frac{dx}{dt}$. The first class consists of methods like PRESCIENT [4] and RNAForecaster [6] that can learn f based only on time series gene-expression input $\left\{x_{t_{0}};\;x_{t_{1}};\;\ldots ;\;x_{t_{T}}\right\}$ without additional steps or consideration of other regulatory inputs [4, 6, 15]. In the process of learning transitions between consecutive time points, these “one-step” methods **implicitly** learn the local derivative (often referred to as “RNA velocity” [16]) ${\left.\frac{dx}{dt}\right|}_{x=x_{t_{m}}}$, as an intermediary to estimating f. One significant issue with these approaches is scalability, and studying meaningfully large dynamical systems (ideally those describing the entire genome) has been too costly in terms of runtime and performance loss [4, 6, 17, 18]. This leads to potential issues with generalizability as regulatory processes operate genome-wide and even small perturbations can have wide-ranging regulatory effects.

The second class consists of “two-step” methods such as Dynamo [3], RNA-ODE [5], and scDVF [12] that aim to alleviate this performance loss by replacing the difficult task of learning f with two separate steps [3, 5, 12]. First, the RNA velocity $\left(\frac{dx}{dt}\right)$ is **explicitly** estimated for each data point in a preprocessing step, using spliced and unspliced transcript counts, and one of many available velocity estimation tools [3, 16, 19–23]. In the next step, the original task of learning f is reduced to learning a vector field from expression-velocity tuples $\left[x_{i},{\left(\frac{dx}{dt}\right)}_{i}\right]$ and a suitable learning algorithm is deployed. Apart from needing additional inputs that may not always be available (for example, spliced and unspliced counts are not available for microarray data), these “two step” methods are also sensitive to the velocity estimation tool used, many of which suffer from a multitude of weaknesses [19]. Still, the Jacobian of the estimated vector field can help inform whether the learned dynamics are biologically meaningful [2, 3, 5, 8].

While the flexibility of both classes of models is helpful in estimating arbitrary dynamics, they are“black-box” methods whose somewhat opaque nature not only makes them prone to overfitting, but it makes it difficult to extract interpretable mechanistic insights about regulatory control [1, 6]. These models are optimized solely to predict RNA velocity or gene expression levels and so the predictions are not explainable in the sense that most cannot be related back to a sparse causal GRN. Another major issue is the scalability of these methods; because of their computational complexity, they have not yet been shown to feasibly scale up to tens of thousands of genes – and definitely not to the entire genome [3, 4, 7, 12–14]. Consequently, most of these methods either restrict themselves to a small set of highly variable genes [4, 6, 7, 12, 14] or resort to dimension-reduction techniques (PCA, UMAP, latent-space embedding, etc.) [3, 4, 7, 13] as a preprocessing step . This leads to certain biological pathways being masked in the dynamics and impedes the recovery of causal GRNs. Finally, these models generally lack a means of incorporating biological constraints and prior knowledge to guide model selection and prevent overfitting [1, 24].

We developed **PHOENIX** (**P**rior-informed **H**ill-like **O**DEs to **E**nhance **N**euralnet **I**ntegrals with e**X**plainability) as a scalable method for estimating dynamical systems governing gene expression through an ODE-based machine learning framework that is flexible enough to avoid model misspecification and is guided by insights from systems biology that facilitate biological interpretation of the resulting models [25, 26]. At its core, PHOENIX models temporal patterns of gene expression using neural ordinary differential equations (NeuralODEs) [27, 28], an advanced computational method commensurate with the scope of human gene regulatory networks – with more than 25,000 genes and 1,600 TFs – and the limited number of samples. We implement an innovative NeuralODE architecture that inherits the universal function approximation property (and thus the flexibility) of neural networks while resembling Hill–Langmuir kinetics (which have been used to model dynamic transcription factor biding site occupancy [10, 29, 30]) so that it can reasonably describe gene regulation by modeling the sparse yet synergystic interactions of genes and transcription factors.

Importantly, PHOENIX operates on the original gene expression space and does not require any dimensionality reduction, thus preventing information loss (especially for lowly-expressed genes that are nonetheless important for cell fate) [4]. This together with the incorporation of user-defined prior knowledge of likely network structure ensures that a trained PHOENIX model is explainable – it not only predicts temporal gene expression patterns, but also encodes an extractable GRN that captures key mechanistic properties of regulation such as activating (and repressive) edges and strength of regulation.

## The PHOENIX model

**Figure 1:**
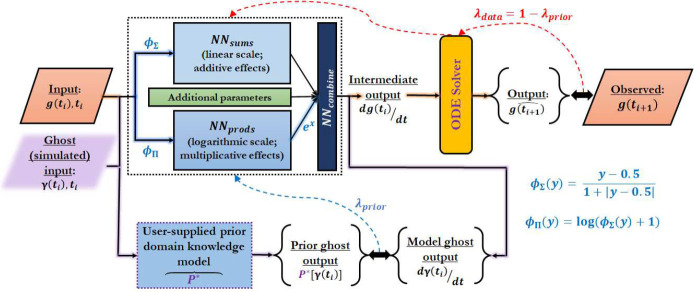
PHOENIX is powered by a NeuralODE engine. Given an expression vector $g\left(t_{i}\right)\in {\mathbb{R}}^{\#\text{genes}}$ at time $t_{i}$, a neural network (dotted rectangle) estimates the local derivative $dg\left(t_{i}\right)/dt$ and an ODE solver integrates this value to predict expression at subsequent time points $\overset{ˆ}{g}\left(t_{i+1}\right)$. The neural network is equipped with activation functions ($\phi_{\Sigma }$ and $\phi_{\Pi }$) that resemble Hill-Langmuir kinetics, and two separate single-layer blocks $\left({NN}_{\mathrm{sums}}\right.$ and ${NN}_{\mathrm{prods}}$) that operate on the linear and logarithmic scales to model additive and multiplicative co-regulation respectively. A third block (${NN}_{\mathrm{combine}}$) then flexibly combines the additive and multiplicative synergies. PHOENIX incorporates two levels of back-propagation to parameterize the neural network while inducing domain knowledge-specific properties; the first (red arrows with weight $\lambda_{\text{data}}$) aims to match the observed data, while the second (blue arrow with weight $\lambda_{\text{prior}}$) uses simulated (ghost) expression vectors $\gamma \left(t_{i}\right)$ to implement soft constraints defined by user-supplied prior models $\left({𝒫}^{*}\right)$ of putative regulatory interactions.

Given a time series gene expression data set, the NeuralODEs of PHOENIX implicitly estimate the local derivative (RNA velocity) at an input data point with a neural network (NN). We designed activation functions that resemble Hill-kinetics and thus allow the NN to sparsely represent different patterns of transcriptional co-regulation by combining separate additive and multiplicative blocks that operate on the linear and logarithmic scales respectively. An ODE solver then integrates the estimated derivative to reconstruct the steps taken from an input $x_{i}$ at time $t_{i}$ to a predicted output ${\hat{x}}_{i+1}$ at time $t_{i+1}$ [27]. The trained neural network block thus encodes the ODEs governing the dynamics of gene expression, and hence encodes the underlying vector field and GRN. An important advantage of incorporating an ODE solver is that we can predict expression-changes for arbitrarily long time intervals without relying on predefined Euler discretizations, as is required by many other methods [4, 12, 18]. We further augmented this framework by allowing users to include prior knowledge of gene regulation in a flexible way, which acts as a domain-knowledge-informed regularizer or soft constraint of the NeuralODE [24] (Figure 1). By combining the mechanism-driven approach of systems biology inspired functional forms and prior knowledge with the data-driven approach of powerful machine learning tools, PHOENIX scales up to full-genome data sets and learns meaningful models of gene regulatory dynamics.

## Neural ordinary differential equations (NeuralODEs)

NeuralODEs [27] learn dynamical systems by parameterizing the underlying derivatives with neural networks:

$$\frac{dx(t)}{dt}=f(x(t),t)\approx {NN}_{\theta }(x(t),t)$$

Given an initial condition, the output at any given time-point can now be approximated using a numerical ODE solver 𝒮 of adaptive step size:

$$\hat{x\left(t_{1}\right)}=x\left(t_{0}\right)+\int_{t_{0}}^{t_{1}}\;{NN}_{\theta }(x(t),t)dt=𝒮\left(x\left(t_{0}\right);\;{NN}_{\theta };\;t_{0};\;t_{1}\right)$$

This is the basic architecture of a NeuralODE [27], and it lends itself to loss functions L (e.g. $\ell_{2}$ loss) of the form:

$$L\left(x\left(t_{1}\right),\hat{x\left(t_{1}\right)}\right)=L(x\left(t_{1}\right),𝒮\left(x\left(t_{0}\right);\;{NN}_{\theta };\;t_{0};\;t_{1}\right)).$$

To perform back-propagation, the gradient of the loss function with respect to all parameters *θ* must be computed, which is done using the adjoint sensitivity method [27]. Building off of the NeuralODE author’s model implementation in PyTorch [28], we made biologically-motivated modifications to the architecture, and incorporated user-defined prior domain knowledge.

## Model formulation and neural network architecture

Most models for co-regulation of gene expression are structured as a simple feedback process [29]. Given that gene regulation can be influenced by perturbations across an entire regulatory network of n genes, the gene expression of all genes $g_{j}(t)$ can have an effect on a specific $g_{i}(t)$ at time point *t*:

$$\frac{dg(t)}{dt}=f_{reg}(g(t))-g(t)$$

where $g(t)={\left\{g_{i}(t)\right\}}_{i=1}^{n},\;f_{\text{reg}}:R^{n}\to R^{n}$, and $f_{\text{reg}}$ is approximated with a neural network. To model additive as well as multiplicative effects within $f_{\text{reg}}$, we used an innovative neural network architecture equipped with activation functions that emulate - and can thus sparsely encode - Hill-kinetics (see Figure 1). The Hill-Langmuir equation H(P) was originally derived to model the binding of ligands to macromolecules [30], and can be used to model transcription factor occupancy of gene regulatory binding sites [10]:

$$H(P)=\frac{P^{\alpha }}{\kappa^{\alpha }+P^{\alpha }}=\frac{(P/\kappa {)}^{\alpha }}{1+(P/\kappa {)}^{\alpha }}=\frac{Y}{1+Y},\;\text{with}\;Y=(P/\kappa {)}^{\alpha },$$

which resembles the softsign activation function $\phi_{\text{soft}}\;(y)=1/(1+|y|)$. For better neural network trainability, however, we shifted it to the center of the expression values. To approximate suitable exponents α, we further log transformed H, since composing additive operations in the log transformed space with a Hadamard exp o function can represent multiplicative effects. Thus,

$$\phi_{\Sigma }(x)=\frac{x-0.5}{1+|x-0.5|},\;\phi_{\Pi }(x)=log\left(\frac{x-0.5}{1+|x-0.5|}+1\right).$$

were employed as activation functions to define two neural network blocks (${NN}_{\mathrm{sums}}$ and ${NN}_{\mathrm{prods}}$), representing additive and multiplicative effects:

$$c_{\Sigma }(g(t))=W_{\Sigma }\phi_{\Sigma }(g(t))+b_{\Sigma }\;c_{\Pi }(g(t))=\exp\circ \left(W_{\Pi }\phi_{\Pi }(g(t))+b_{\Pi }\right).$$

The concatenated vectors $c_{\Sigma }(g(t))⊕c_{\Pi }(g(t))$ served as input to a third block ${NN}_{\mathrm{combine}}$ (with weights $W_{\cup }\in R^{n\times 2m}$) that flexibly combined these additive and multiplicative effects. We found that a single linear layer was sufficient for this purpose. To simplify the representation of steady state for genes without upstream transcription factors $\left(\frac{dg_{i}(t)}{dt}=0,\forall t\right)$, we introduced gene specific parameters $v\in R^{n}$. Accordingly, the output derivative for each gene i was multiplied with $ReLU\left(v_{i}\right)=max\left\{\left(v_{i},0\right)\right\}$. We expressed this using the Hadamard product (⊙) of the previous output and the elementwise ReLU of v:

$$\frac{\hat{dg(t)}}{dt}=\mathrm{ReLU}(v)⊙[W_{\cup }\left\{c_{\Sigma }(g(t))⊕c_{\Pi }(g(t))\right\}-g(t)].$$

The trainable parameters $\theta =\left(W_{\Sigma },W_{\Pi },b_{\Sigma },b_{\Pi },W_{U},v\right)$ were learned based on observed data and prior domain knowledge (details in Supp. Methods 1).

## Structural domain knowledge incorporation

One challenge we found in interpreting PHOENIX is that NeuralODEs have multiple solutions [31], of which many are inconsistent with our understanding of the process by which specific transcription factors (TFs) regulate the expression of other genes within the genome. Most solutions accurately represent gene-gene correlations, but do not necessarily reflect biologically established TF-gene regulation processes. Inspired by recent developments in physics-informed deep learning [24], we introduced biologically-motivated soft constraints to regularize the search for a parsimonious approximation. We started with the NeuralODE prediction for the gene expression vector:

$$\hat{g\left(t_{1}\right)}=g\left(t_{0}\right)+\int_{t_{0}}^{t_{1}}\;\mathrm{ReLU}(v)⊙[W_{\cup }\left\{c_{\Sigma }(g(t))⊕c_{\Pi }(g(t))\right\}-g(t)]dt\;\;=𝒮(g\left(t_{0}\right);\mathrm{ReLU}(v)⊙\left[W_{\cup }\left\{c_{\Sigma }(g(t))⊕c_{\Pi }(g(t))\right\}-g(t)\right];\;t_{0};\;t_{1})$$

We observed that the unregularized PHOENIX provides an observed gene expression-based approximation for the local derivative $\frac{dg(t)}{dt}$, but often we have additional structural information available about which TFs are more likely to regulate certain target genes. Hence, one could also formulate a domain knowledge-informed ${𝒫}^{*}(g(t))$ that is a prior-based approximation:

$$\underset{\text{PHOENIX}\;\text{(based}\;\text{on}\;\text{observed}\;\text{gene}\;\text{expression}\;\text{data)}}{\underbrace{\mathrm{ReLU}(v)⊙\left[W_{\cup }\left\{c_{\Sigma }(g(t))⊕c_{\Pi }(g(t))\right\}-g(t)\right]}}\approx \frac{dg(t)}{dt}\approx \underset{\text{prior-based}}{\underbrace{{𝒫}^{*}(g(t))}}.$$

By promoting our NeuralODE to flexibly align with such structural domain knowledge, we automatically searched for biologically more realistic models that still explained the observed gene expression data. To this end, we designed a modified loss function ${ℒ}_{\text{mod}}$ that incorporated the effect of prior model ${𝒫}^{*}$ using a set of K randomly generated expression vectors ${\left\{\gamma_{k}\in R^{n}\right\}}_{k=1}^{K}$. This induced a preference for consistency with prior domain knowledge.


$$\begin{array}{l} \;{ℒ}_{\mathrm{mod}}\left(g\left(t_{1}\right),\hat{g\left(t_{1}\right)}\right)\\ =\lambda \overset{\text{loss}\;\text{based}\;\text{on}\;\text{matching}\;\text{observed}\;\text{gene}\;\text{expression}\;\text{data}}{\overbrace{L[g\left(t_{1}\right),𝒮(g\left(t_{0}\right);\;\mathrm{ReLU}(v)⊙[W_{\cup }\left\{c_{\Sigma }(g(t))⊕c_{\Pi }(g(t))\right\}-g(t)];\;t_{0};\;t_{1})]}}\\ \;+(1-\lambda )\underset{\text{loss}\;\text{based}\;\text{on}\;\text{matching}\;\text{prior}\;\text{model}}{\underbrace{\frac{1}{K}\sum_{k=1}^{K}\;L[{𝒫}^{*}\left(\gamma_{k}\right),\mathrm{ReLU}(v)⊙[W_{\cup }\left\{c_{\Sigma }\left(\gamma_{k}\right)⊕c_{\Pi }\left(\gamma_{k}\right)\right\}-\gamma_{k}]]}} \end{array}$$

Here, λ is a tuning parameter for flexibly controlling how much weight is given to the prior-based optimization, which we tuned with cross-validation, and $L[x,\hat{x}]$ is the primary loss function, set to the $L=\ell_{2}$ loss in our experiments.

While our modeling framework is flexible regarding the nature of the prior model ${𝒫}^{*}$, we incorporated a simple linear model, a common choice for chemical reaction networks or simple oscillating physical systems [32]. We used the adjacency matrix A of likely network structure based on prior domain knowledge (e.g. experimentally validated interactions, motif map of promoter targets, etc.) with known activating and repressive edges set to +1 and −1 respectively. For cases where the sign (activating/repressive) was unknown, we randomly assigned +1 or −1 with uniform probability.


$${𝒫}^{*}\left(\gamma_{k}\right)=A\cdot \gamma_{k}-\gamma_{k}=(A-I)\cdot \gamma_{k}$$

## Results

We demonstrate the utility of PHOENIX for estimating gene expression dynamics by performing a series of *in silico* benchmarking experiments, where PHOENIX exceeds even the most optimistic performance of popular black-box RNA dynamics estimation methods. We demonstrate the scalability of PHOENIX by applying it to genome-scale breast cancer samples ordered in pseudotime and investigate how scaling to the complete data set improves a representation of key pathways. Finally, we apply PHOENIX to yeast cell cycle data to show that it can capture oscillatory dynamics by flexibly deviating from Hill-like assumptions when necessary.

### PHOENIX accurately and explainably learns temporal evolution of *in silico* dynamical systems

We began our validation studies with simulated gene expression time-series data so that the underlying dynamical system that produced the system’s patterns of gene expression was known. We adapted SimulatorGRN [29, 33] to generate time-series expression data from two synthetic *S. cerevisiae* gene regulatory systems (SIM350 and SIM690, consisting of 350 and 690 genes respectively). The activating and repressive interactions in each *in silico* system was used to synthesize noisy expression “trajectories” for each gene across multiple time-points (see Methods 1.1 and 1.2). We split up the trajectories into training (88%), validation (6% for hyperparameter tuning), and testing (6%), and compared PHOENIX predictions on the test set against the “known”/ground truth trajectories (detailed results in Supp. Results 1). Since PHOENIX uses user-defined prior knowledge as a regularizer, we also corrupted the prior model at a level commensurate with the “experimental” noise level (see Supp. Methods 4.2), reflecting the fact that transcription factor-gene binding is itself noisy.

**Figure 2:**
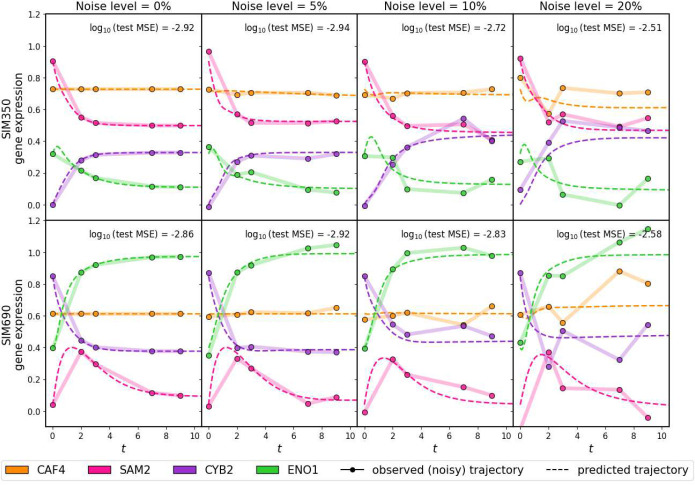
We applied PHOENIX to simulated gene expression data originating from two different *in silico* dynamical systems SIM350 (top row) and SIM690 (bottom row) that simulate temporal expression of 350 and 690 genes respectively. Each simulated trajectory consisted of five time-points (*t* = 0, 2, 3, 7, 9) and was subjected to varying levels of Gaussian noise $\left(\frac{\text{noise}\;\sigma }{\text{mean}}=0\%,\;5\%,\;10\%,\;20\%\right)$. Since PHOENIX uses a user-defined prior network model as a regularizer, we also corrupted the prior-models up to an amount commensurate with the noise level. For each noise setting we trained PHOENIX on 140 of these “observed” trajectories and validated on 10. The performance on the validation trajectories was used to determine the optimal value of λ_prior_. We then tested the trained model (with the optimal choice of λ_prior_) on 10 new test set trajectories. We display both observed and predicted test set trajectories for four arbitrary genes in both SIM350 and SIM690, across all noise settings. We display the mean squared error (MSE) between the predictions and the 10 test set trajectories pre-noise.

We found that PHOENIX accurately learned the temporal evolution of the SIM350 and SIM690 data sets (Figure 2) and was able to recover the **true** test set trajectories (that is, test set trajectories pre-noise) with a reasonably high accuracy even when the training trajectories and prior knowledge model had included high levels of noise. Furthermore, the shapes of the predicted trajectories (and hence the predicted steady state levels) obtained from feeding initial values (expression at *t* = 0) into the trained model remained robust to noise, suggesting that a trained PHOENIX model could be used to estimate the temporal effects of cellular perturbations.

Since the primary prediction engine of PHOENIX is a NeuralODE, we wanted to benchmark its performance relative to “out-of-the-box” (OOTB) NeuralODE models (such as RNAForecaster [6]) to understand the contributions of our modifications to the NeuralODE architecture. We tested a range of OOTB models (activation functions: ReLU, sigmoid, and tanh) where we adjusted the total number of trainable parameters to be similar to that of PHOENIX (see Supp. Methods 3.2). Because PHOENIX uses a domain prior of likely gene-regulation interactions in its optimization scheme, we also tested a version (PHX_0_) where the weight of the prior was set to zero (λ_prior_ = 0). For each of SIM350 and SIM690, we observed that PHOENIX outperformed OOTB NeuralODEs on the test set in noiseless settings (Supp. Fig. 1 and Supp. Table 2). When we added noise, the PHOENIX models still generally outperformed the OOTB models, especially in the larger SIM690. The validation MSEs were more comparable between all the models in the high noise setting in SIM350. The consistently strong performance of PHOENIX suggests that using a Hill-kinetics inspired NeuralODE architecture better captures the dynamics of the regulatory process, in part because it models the binding kinetics of transcription factor-gene interactions.

In terms of contribution of the prior to PHOENIX’s performance, we observed that PHOENIX was generally outperformed by PHX_0_, its unregularized version (Supp. Fig. 1 and Supp. Table 2). However, given that the prior can be interpreted as soft biological constraints on the estimated dynamical system [24], an important question is whether PHX_0_ (as well as OOTB models) makes accurate temporal predictions by correctly learning elements of the causal biology, or whether the lack of prior information results in an alternate learned representation of the dynamics, which - despite predicting these particular trajectories well - does not explain the true biological regulatory process.

To this end, we recognized that the parameters of a trained PHOENIX model encode an estimate of the ground-truth gene regulatory network (GRN) that causally governs the system’s evolution over time. We therefore inferred encoded GRNs from trained PHOENIX models and compared them to the ground truth networks *GRN*_350_ and *GRN*_690_ used to synthesize SIM350 and SIM690 respectively (see Supp. Methods 2). Given PHOENIX’s simple NeuralODE architecture, we were able to develop a GRN inference algorithm that could predict edge existence, direction, strength, and sign, using just model coefficients, without any need for time consuming sensitivity analyses (unlike other approaches [2, 12]). For comparison, we wanted to extract GRNs from the most predictive OOTB models; given their black-box nature, OOTB model GRNs had to be obtained via sensitivity analyses (see Supp. Methods 3.2).

**Figure 3:**
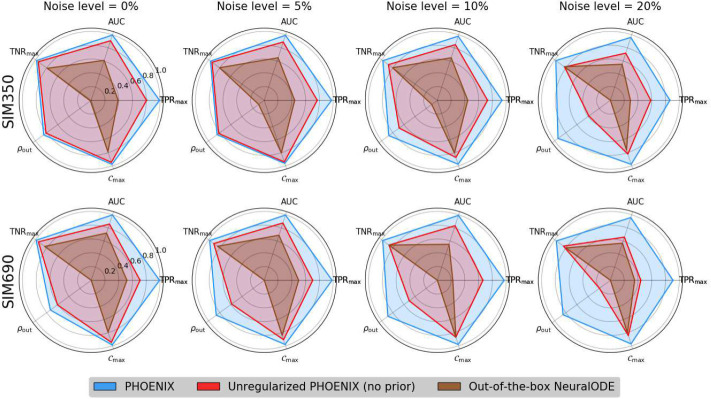
We extracted encoded GRNs from the trained PHOENIX models and the best performing out-of-the-box NeuralODE models, for both *in silico* dynamical systems SIM350 (top row) and SIM690 (bottom row) across all noise settings. We compared these GRN estimates to the corresponding ground truth GRNs used to formulate SIM350 and SIM690, and obtained AUC values as well as out-degree correlations (*ρ*_out_). We also reverse-engineered a metric (*C*_max_) to inform how sparsely PHOENIX had inferred the dynamics (see Supp. Methods 2). Furthermore, we used these *C*_max_ values to obtain optimal true positive and true negative rates (TPR_max_ and TNR_max_) that were independent of any cutoff value, allowing us to compare between “best possible” networks across all settings.

We compared inferred and ground truth GRNs in terms of several metrics, including edge recovery, out-degree correlations, and induced sparsity. We obtained near-perfect edge recovery for PHOENIX (AUC= 0.96 – 0.99) as well as high out-degree correlations across all noise settings (Figure 3 and Supp. Table 3). Most notably, we observed that PHOENIX predicted dynamics in a more robustly explainable way than PHX_0_ and the OOTB models. We measured induced sparsity by reverse engineering a metric *C*_max_ based on maximizing classification accuracy (see Supp. Methods 2), and found that PHOENIX resulted in much sparser dynamics than PHX_0_ (Supp. Table 4). To further assess this phenomenon, we computed the estimated model effect between every gene pair in SIM350, and compared these values between PHOENIX and PHX_0_. We found that that the incorporation of priors helped PHOENIX identify core elements of the dynamics, and predict gene expression patterns in a biologically parsimonious manner (Supp. Fig. 2).

Since the inclusion of such static prior knowledge greatly increased the explainability of the inferred dynamics, we also investigated how explainability was affected by misspecification of the prior. In our *in silico* experiments, we had randomly corrupted (misspecified) the prior by an amount commensurate with the noise level (see Supp. Methods 4.2). We compared network representations of these misspecified prior constraints to GRNs extracted from the PHOENIX models that used these very priors. We found that PHOENIX was able to appropriately learn causal elements of the dynamics beyond what was encoded in the priors (Supp. Table 1). This suggests that even though the user-defined priors enhance explainability, PHOENIX can deviate from them when necessary, and learn regulatory interactions from just the data itself.

### PHOENIX exceeds the most optimistic performances of current black-box methods *in silico*

Having established PHOENIX models as both predictive and explainable, we compared its performance to other existing methods for gene expression ODE estimation *in silico* (Table 1). As discussed earlier, these can be placed into two groups based on the input data. The “one-step” methods estimate dynamics by directly using expression trajectories; these include RNAForecaster [6] (which is an out-of-the-box NeuralODE), and PRESCIENT [4], among others [14, 15]. PHOENIX is more similar to these methods.

“Two-step” methods such as Dynamo [3], RNA-ODE [5], and scDVF [12] estimate dynamics by first reconstructing RNA velocity using inputs such as spliced and unspliced mRNA counts and then estimating a vector field mapping expression to velocity. To avoid the need for uncommon input data types and to also emulate the theoretically optimal performance in the first step of these “two-step” velocity-based methods, we used the noiseless ground truth velocities as input into their second step since the true velocities were known in our *in silico* experiments (see Supp. Methods 3.1). Further, we used the validation set to optimize key hyperparameters of all the methods (Table 1, right-most column) before finally testing predictive performance on expression values from held-out trajectories. Most of the methods also provide a means for extracting a gene network which we used to evaluate each method’s explainability (see Supp. Methods 3.3).

In these comparisons, we found that the “one-step” trajectory-based methods generally yielded better predictions than the “two-step” velocity-based methods (although Dynamo sometimes achieved performance compared to the single step methods). This makes sense since trajectory-based methods are optimized to predict gene expression trajectories while velocity-based methods predict trajectories by first optimizing RNA velocity estimations [1]. Overall, PHOENIX outperformed even the optimistic versions of the black-box methods by large margins both in terms of predicting gene-expression (Supp. Table 2) and explainability (based on consistency with the ground truth network Supp. Table 3). We found that Dynamo was generally the most explainable competing method but that, in some settings, RNA-ODE and scDVF were more explainable. Finally, we found that the dynamics estimated by PHOENIX were much sparser than any other method, and that this sparsity remained fairly insensitive to noise levels (Supp. Table 4).

Further ODE estimation approaches (not included in our experiments) and their functionalities are discussed in Supp. Table 5. Code for performing such methodological benchmarks are included with the PHOENIX release [34].

### PHOENIX predicts temporal evolution of yeast cell-cycle genes in an explainable way

We tested PHOENIX using an experimental data set [35] from cell-cycle synchronized yeast cells, consisting of two technical replicates of expression values for 3551 genes across 24 time points (see Methods 2.1 for data processing).

Since there were two technical replicates (or trajectories), one approach for training PHOENIX was to use one replicate for training and the other for validation. However, given the extreme similarity between the two replicates in terms of gene expression values across all 24 times points, this would have led to artificially good performance on the validation trajectory. Instead of splitting by trajectories here, we used an alternate strategy and split the data based on transition pairs (see Methods 2.2), splitting the 46 transition pairs into training (40, 86%), validation (3, 7%), and test (3, 7%). For the domain prior we used a simplistic adjacency-matrix-based prior model derived from a motif map of promoter targets for each of the 3551 genes (see Methods 2.2). We tuned the prior weight (to λ_prior_ = 0.10) using the validation set to induce higher explainability by promoting a sparse GRN structure.

**Figure 4:**
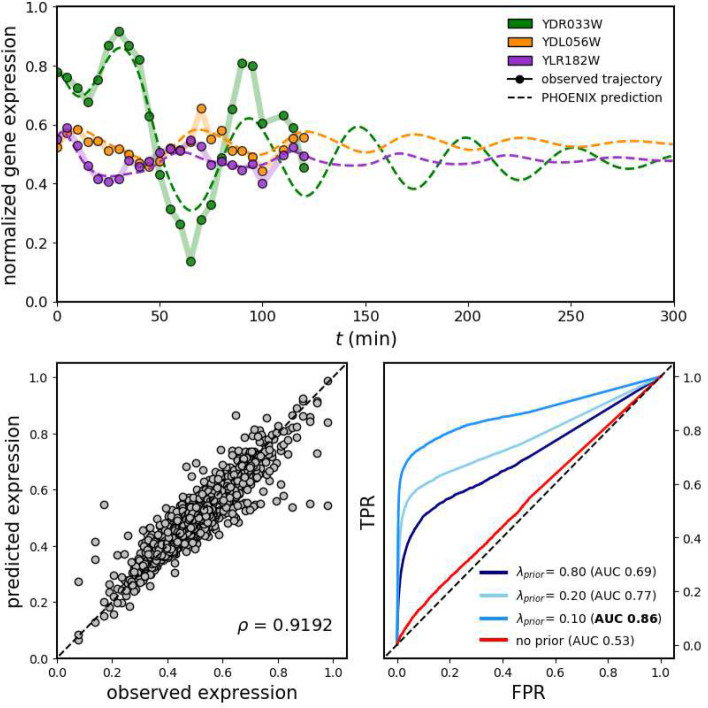
(top) We applied PHOENIX (λ_*prior*_ = 0.10) to 2 technical replicates of gene expression of 3551 genes each, collected across 24 time points in a yeast cell-cycle time course [35]. We trained on 40 transition pairs, used 3 for validation, and tested predictive accuracy on the remaining 3. We display both observed and predicted trajectories for 3 arbitrary genes, where the predicted trajectories are extrapolations into future time points based on just initial values (gene-expression at *t* = 0). (bottom-left) We correlated observed versus predicted expression levels of all 3551 genes for the 3 expression vectors in the test set; *ρ* = 0.919 implying *R*^2^ = 0.844. (bottom-right) We tested the explainability of the learned dynamics by comparing encoded GRNs retrieved from a series of trained models (of varying prior-dependencies) against ChIP-chip data [37] to obtain ROC curves. The λ_prior_ = 0.10 model was the one chosen based on validation set MSE (see Supp. Methods 1).

PHOENIX was able to learn the temporal evolution of gene expression across the yeast cycle; it was able to explain over 84% of the variation in the test set (Figure 4, bottom-left). Notably, when we visualized the estimated dynamics by extrapolating from just initial values (expression at *t* = 0), we found that PHOENIX plausibly predicted continued periodic oscillations in gene expression, even though the training data consisted of only two full cell cycles (Figure 4, top). The amplitude of the predicted trajectories dampened across time points, which is expected given that yeast array-data tends to exhibit underdamped harmonic oscillation during cell division possibly reflecting de-synchronization of the yeast cells [36]. This performance on oscillatory dynamics is indicative of the high flexibility of PHOENIX, which inherits the universal function approximation property from NeuralODEs, allowing it to deviate from Hill-like assumptions when necessary, while still remaining explainable due to the integration of prior knowledge.

To test the biological explainability of the learned dynamical system, we extracted the encoded GRN from the trained PHOENIX model (with optimal λ_prior_ = 0.10 as determined by the validation set) and compared to a validation network of ChIP-chip transcription factor (TF) binding data [37]. PHOENIX had impressive accuracy in predicting TF binding (AUC = 0.86), indicating that it had learned transcription factor binding information in the process of explaining temporal patterns in expression (Figure 4, bottom-right). In the absence of any prior-knowledge (λ_prior_ = 0), the explainability was poor, highlighting the importance of such knowledge-based guidance in black-box models [24, 25].

Similar to the *in silico* experiments, we saw that PHOENIX’s ability to predict TF binding was greater than that obtained by comparing just the prior to the validation data (Supp. Table 1). This suggested that PHOENIX had used the prior knowledge of cell cycle progression as a starting point to anchor the dynamics, and then used the data itself to learn improved regulatory rules.

### PHOENIX infers genome-wide dynamics of breast cancer progression and identifies central pathways

Although there are a number of tools for inferring dynamics of regulatory networks, most do not scale beyond a few hundreds of genes, falling far short of the 25,000 genes in the human genome (Table 1). Given the performance improvements we saw that were driven by PHOENIX’s use of soft constraints, we wanted to test whether PHOENIX could be extended to human-genome scale networks. Due to the dearth of longitudinal human studies with genome-wide expression measurements, we used data from a cross-sectional breast cancer study (GEO accession GSE7390 [38]) consisting of microarray expression values for 22000 genes from 198 breast cancer patients, and ordered these samples in pseudotime. For consistency in pseudotime ordering, we reused a version of this data that was already preprocessed and ordered (using a random walk based pseudotime approach) in the PROB paper [8].

We limited our analysis to the genes that had measurable expression and appeared in our regulatory prior, and obtained a single pseudotrajectory of expression values for *n*_*g*_ = 11165 genes across 186 patients, each at a distinct pseudo-timepoint. To explore whether PHOENIX’s performance depends on the size of the data set, we created pseudotrajectories for *n*_*g*_ = 500, 2000, and 4000 genes by subsetting the data set to its *n*_*g*_ most variable genes. Similar to the yeast example, we split up the 185 transition pairs into training (170, 90%), validation (8, 5%), and test (7, 5%). For the domain prior network, we again used a simplistic prior model derived from a motif map of promoter targets (see Methods 3.2), and tuned λ_prior_ using the validation set.

**Figure 5:**
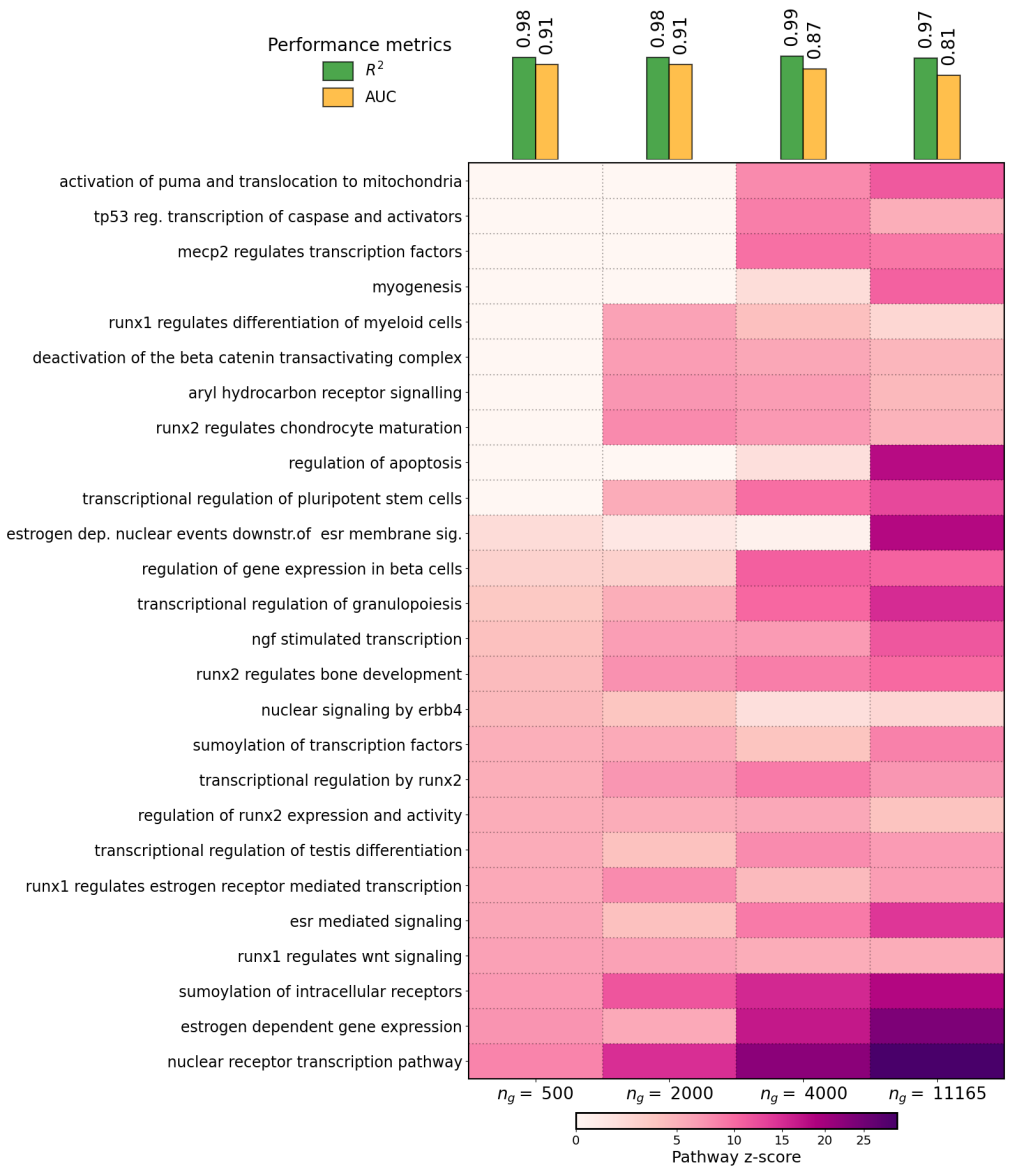
(top panel) We applied PHOENIX to a pseudotrajectory of 186 breast cancer samples (ordered along subsequent “pseudotimepoints”) consisting of *n*_*g*_ = 11165 genes [38]. We trained on 170 transition pairs, used 8 for validation (to tune λ_prior_), and tested predictive accuracy on the remaining 7. We also repeated the analysis on smaller subsets of genes *n*_*g*_ = 500, 2000, 4000, where we subsetted the full trajectory to only the *n*_*g*_ most variable genes in the pseudotrajectory. We display both the predictive performance (*R*^2^ on the test set) and the explainability performance (AUC from comparing encoded GRNs from trained models against a ChIP-seq validation network [39]). (main panel) We used the trained PHOENIX models to extract permutation-based influence scores for pathways in the Reactome database [51] (see Methods 3.3 and 3.4), and visualized influence scores for a collection of the most central pathways. See Supp. Table 6 for detailed results.

For each pseudotrajectory of size *n*_*g*_, we fit a separate PHOENIX model and calculated variation explained in the test set. We observed very impressive predictive performance, with test set *R*^2^ values in the 97% - 99% range (Figure 5, top). We found that even though PHOENIX’s computational cost increased as we scaled to *n*_*g*_ = 11165 genes, the cost was not excessive even at this genome-scale (see Supp. Table 7). It is noteworthy that this type of feasible scalability to *n*_*g*_ > 10^4^ genes is unprecedented in tools for estimating gene regulatory dynamical systems; other methods either focus on a subset of highly variable genes [6, 7, 12, 14] or model dynamics in a less interpretable, lower dimensional PCA/UMAP/latent space [3, 4, 13].

Next, we investigated PHOENIX’s ability to identify biologically relevant and actionable information regarding gene regulation in breast cancer. First, we tested the performance of the learned dynamical system to reconstruct a gene regulatory network and predict TF-gene interactions. While the ground truth GRN is unknown, we can estimate performance by comparing a validation network of experimental ChIP-chip binding information [39] to a subnetwork of the encoded GRN of a trained PHOENIX model. We found good alignment between the two GRNs (AUC = 0.81 – 0.91) even when we scaled up to *n*_*g*_ = 11165 genes (Figure 5). It is important to note that the PHOENIX-based concordance with experimental data was generally greater than that obtained by comparing just the prior knowledge to the validation network (Supp. Table 1), indicating that PHOENIX was improving upon the GRN suggested by the prior knowledge, in addition to learning a dynamical model.

To better understand the benefits of PHOENIX’s scalability, we investigated how estimating regulatory dynamics based on a subset of only the *n*_*g*_ most variable genes can alter the perceived importance of individual genes to the regulatory system in question. We reasoned that a model trained on all assayed genes should reconstruct biological information better than those that are restricted to a subset of genes [6, 12, 14]. First, we performed a gene-level analysis by perturbing *in silico* the PHOENIX-estimated dynamical system from each value of *n*_*g*_ (500, 2000, 4000, 11165). This yielded “influence scores” representing how changes in initial (*t* = 0) expression of each gene affected subsequent (*t* > 0) predicted expression of all other genes (see Methods 3.3). As might be expected, the influence scores grew increasingly more concordant with centrality measures in the ChIP validation network, consistent with the key roles played by transcription factor genes in large GRNs (Supp. Table 7).

We observed that highly variable genes with known involvement in breast cancer (such as WT1 [40], ESR1 [41], AR [42], and FOXM1 [43]) were generally influential across all values of *n*_*g*_ (Supp. Fig. 3). It is interesting to note that both WT1 and FOXM1 were very influential in the *n*_*g*_ = 500 system, but their score drops in the full genome (*n*_*g*_ = 11165) system. This is likely due to the way in which we constructed the smaller subsets of the whole genome – by selecting the most variable genes. One would expect that the most variable transcription factor genes falling within any subset would be highly correlated in expression with other genes falling in the same set, and that the overall effect would be diluted by adding more - potentially uncorrelated - genes to the system. It is more interesting that genes missing in the smaller subsets (due to low expression variability) were identified as central to the dynamics in the full (*n*_*g*_ = 11165) system. Among these genes, we can find some encoding cancer-relevant transcription factors such as E2F1 [44, 45] and CTCF [46], members of the TP53 family (TP73 [47, 48]), DNA methyltransferase enzymes (DNMT1 [49]), and members of the KLF family [50].

We found that the more computationally manageable systems (*n*_*g*_ = 500, *n*_*g*_ = 2000) yielded an incomplete picture of gene-level influences, since the the method used in constructing these subsets hinders the mechanistic explainability of the resulting regulatory model. Certain genes exhibit relatively low variability in expression but are still central to disease-relevant genome-level dynamics; compared to methods that exclude such genes to make computation tractable [4, 6, 12], PHOENIX can correctly identify such as central because of its ability to model subtle but important genome-scale dynamics.

Finally, we performed a pathway-based functional enrichment analysis by translating these gene influence scores to pathway influence scores using permutation tests on the Reactome pathway database [51] (see Methods 3.4). Not surprisingly, the dynamical systems with fewer genes missed many pathways known to be associated with breast cancer that were identified as over-represented in the genome-scale (*n*_*g*_ = 11165) system (Figure 5 and Supp. Table 6). Notably, the pathways missed in the smaller networks include apoptosis regulation (a hallmark of cancer [52]), estrogen-related signalling (whose role in breast cancer is well documented [53]), regulation of beta cells (relevant for immune processes [54]), and TP53 regulation of caspases (relevant to apoptosis control in tumors [55]).

In a parallel analysis testing for functional enrichment of GO biological process terms, we again found the smaller systems to overlook important pathways that were clearly influential in the genome-scale analysis; these included developmental processes associated with epithelial tumor development, germ cell development (possibly linked to the regulation of cancer testis antigens), and a wide array of RNA metabolism processes that are increasingly recognized as being significant to breast cancer development [56] (Supp. Fig. 4). Similarly, for GO molecular function terms, the smaller gene subsets missed key processes such as estrogen receptor binding (Supp. Fig. 5).

These results clearly demonstrate the importance of scalable methods such as PHOENIX that can model genome-wise dynamics. Our reduced gene sets from which we built the smaller PHOENIX models consisted of the 500, 2000, or 4000 most variable genes. These gene sets likely consist of variable genes that are correlated with each other, meaning that we are sampling only a portion of the biological processes driving the temporal changes in breast cancer; the full picture only emerges when looking at regulatory processes across the spectrum of genes that can contribute. Alternative approaches, such as concentrating on specific pathways, risk introducing self-fulfilling biases in the discovery process. Similarly, methods that use low-dimensional embedding to reduce the complexity of modeling dynamics risk obscuring losing valuable, biologically relevant insights. PHOENIX’s scalability offers the best potential for discovery of interpretable insights with high explainability relative to the phenotypes under study.

## Discussion

Given the importance of regulatory networks and their dynamics, there has been a tremendous interest in inferring and modeling their physical and temporal behavior. The use of NeuralODEs represents an extremely promising technology for inferring such networks, but so far, attempts to implement NeuralODE-based network modeling have encountered significant problems, not the least of which has been their inability to scale to modeling genome-wide dynamics in a biologically explainable manner.

PHOENIX represents an important new methodological extension to the NeuralODE framework that is not only scaleable to the full human genome, but also biologically well interpretable and able to capture explicitly both additive as well as multiplicative ways in which transcription factors cooperate in regulating gene expression. For a simplified analysis, the underlying gene regulatory network can also be extracted from a learned model and compared with experimental evidence. An optional feature of PHOENIX that contributes significantly to its explainability is that it can be guided by (structural) domain knowledge. Notably, PHOENIX also remains flexible to deviate from domain knowledge when necessary, and learn novel insights consistent with the training data.

The predictive accuracy, scaleabilty, flexibility, and biological explainability can be attributed primarily to two things. First, our novel NeuralODE architecture that includes the use of Hill-like activation functions for capturing the kinetic properties of molecular binding provides a massive advantage in terms of predictive power. And second, the introduction of soft constraints based on prior knowledge of putative network structure leads to a scalable and biologically explainable estimate of the underlying dynamics.

Using simulated data we have shown that PHOENIX outperforms other models for inferring regulatory dynamics (including other NeuralODE-based models), particularly in the presence of experimental noise. Also, an application to data from the yeast cell cycle elucidates PHOENIX’s flexibility in modelling arbitrary dynamics. More importantly, PHOENIX is the only NeuralODE method capable of extending its modeling to capture genome-scale regulatory processes. Using data from breast cancer patients organized in pseudotime we illustrate not only the ability of PHOENIX to faithfully model genome-scale networks, but also demonstrate the power of extending regulatory modeling to capture seemingly subtle but biologically important regulatory processes.

## Methods

### 1 Testing on simulated data

#### 1.1 Defining a ground truth dynamical system

We created a ground truth gene regulatory network (GRN) by sampling from S. cerevisiae (yeast) regulatory networks obtained from the SynTReN v1.2 supplementary data in simple interaction format (SIF) [57]. The SynTReN file provides a directional GRN containing 690 genes and 1094 edges with annotations (activating vs repressive) for edge types; we defined this GRN to be ground truth network *G*_690_. To obtain *G*_350_, we sampled a subnetwork of 350 genes and 590 edges from *G*_690_. We used the connectivity structure of *G*_350_ and *G*_690_, to define systems of ODEs (SIM350 and SIM690) with randomly assigned coefficients. This entire pipeline was executed using SimulatorGRN [33], a framework used extensively by the R/Bioconductor package dcanr [29]. Please see Supp. Methods 4.1 for futher ODE formulation details from SimulatorGRN.

#### 1.2 Simulating time series gene expression data

For each n∈{350,690}, we used the ground truth dynamical system SIM n to generate expression vectors $g(t)\in R^{n}$, across time points t. We started by i.i.d. sampling 160 standard uniform $R^{n}$ vectors to act as initial (t=0) conditions. We used these initial conditions to integrate SIMn and obtain 160 expression trajectories across t∈T={0,2,3,7,9} using R’s desolve package: ${\left\{{\left\{g(t{)}_{i}\right\}}_{t\in T}\right\}}_{i=1}^{160}$. We used only five time points to emulate potential scarcities of time-series information in real data sets, while the range t=0 to 9 generally covered the transition from initial to steady state. Lastly, we added Gaussian noise vectors $\varepsilon (t,\sigma {)}_{i}\overset{i.i.d}{∼}𝒩\left(0,\sigma^{2}I\right)$ of varying σ to get noisy data sets: ${\left\{{\left\{{\left\{g(t{)}_{i}+\varepsilon (t,\sigma {)}_{i}\right\}}_{t\in T}\right\}}_{i=1}^{160}\right\}}_{\sigma \in S}$. Since the average simulated expression value was ≈ 0.5, using $\sigma \in S=\left\{0,\frac{1}{40},\frac{1}{20},\frac{1}{10}\right\}$ corresponded roughly to average noise levels of 0%, 5%, 10%, 20%.

#### 1.3 Model setup for training and testing

For each simulation scenario, there were 160 simulated trajectories, out of which we used 140 (88%) for training, 10 (6%) for validation (hyperparameter tuning) and 10 (6%) for testing. We provide some details on PHOENIX implementation (e.g. training strategy, prior incorporation, etc) in Supp. Methods 1, and include finer technicalities (e.g. exact learning rates) in our GitHub repository [34]. For prior domain knowledge model we used the simple linear model: ${𝒫}^{*}\left(\gamma_{k}\right)=A^{\sigma \%}\cdot \gamma_{k}-\gamma_{k}$, where we chose $A^{\sigma \%}$ to be noisy/corrupted versions of the adjacency matrices of ground truth networks $G_{350}$ and $G_{690}$ (details in Supp. Methods 4.2). We set activating edges in $A^{\sigma \%}$ to +1 and repressive edges to −1. To validate explainability we extracted GRNs from trained models, and compared to ground truth $G_{350}$ and $G_{690}$ for existence of edges, out-degree correlations, and induced sparsity (details in Supp. Methods 2).

### 2 Testing on experimental yeast cell cycle data

#### 2.1 Data processing and normalization

GPR files were downloaded from the Gene Expression Omnibus (accession GSE4987 [35]), and consisted of two dye-swap technical replicates measured every five minutes for 120 minutes. Each of two replicates were separately ma-normalized using the maNorm() function in the marray library in R/Bioconductor [58]. The data were batch-corrected [59] using the ComBat() function in the sva library [60] and probe-sets mapping to the same gene were averaged, resulting in expression values for 5088 genes across fifty conditions. Two samples (corresponding to the 105 minute time point) were excluded for data-quality reasons, as noted in the original publication, and genes without motif information were then removed, resulting in a expression data-set containing 48 samples (24 time points in each replicate) and 3551 genes. The expression values were then normalized to be between 0 and 1.

#### 2.2 Model setup for training and testing

Given the extreme similarity between the two replicates in terms of gene expression values, the approach of using one replicate for training and the other for validation would have led to artificially good performance on the validation trajectory. Instead, we noted that the data set contained 46 different transition pairs, where a transition pair consists of two consecutive expression vectors in the data set $\left(g\left(t_{i}\right),g\left(t_{i+1}\right)\right)$. We split these 46 transition pairs into training (40, 86%), validation (3, 7%), and test (3, 7%). We provide some details on PHOENIX implementation (e.g. training strategy, prior incorporation, etc) in Supp. Methods 1, and include finer technicalities (e.g. learning rate schedule, initialization scheme, etc) in our GitHub repository [34].

For prior domain knowledge model we used the simple linear model: ${𝒫}^{*}\left(\gamma_{k}\right)=A\cdot \gamma_{k}-\gamma_{k}$. We based our choice of A on the regulatory network structure of a motif map, similar to that used in other methods, such as PANDA [26]. We downloaded predicted binding sites for 204 yeast transcription factors [37]. These data include 4360 genes with tandem promoters. 3551 of these genes are also covered on the yeast cell cycle gene expression array. 105 total transcription factors in this data set target the promoter of one of these 3551 genes. The motif map between these 105 transcription factors and 3551 target genes provides the adjacency matrix A of 0s and 1s, where we randomly assigned each of the 1 s to either +1 (activating) or −1 (repressive).

We used ChIP-chip data from Harbison et al. [37] to create a network of TF-target interactions, and used this as a validation network to test explainability. The targets of transcription factors in this ChIP-chip data set were filtered using the criterion *p* < 0.001. We calculated AUC values by comparing the encoded GRN retrieved from the trained models (see Supp. Methods 2) to the validation network.

### 3 Testing on breast cancer pseudotime data

#### 3.1 Data procurement and psuedotime ordering

The original data set comes from a cross-sectional breast cancer study (GEO accession GSE7390 [38]) consisting of microarray expression values for 22000 genes from 198 breast cancer patients, which we aimed to sort along a pseudotime axis. We noted that the same data set was also used in the PROB [8] paper. PROB is a GRN inference method that infers a random-walk based pseudotime to sort cross-sectional samples and reconstruct the GRN. For consistency and convenience in pseudotime inference, we obtained the same version of this data that was already preprocessed and sorted by PROB. We normalized the expression values to be between 0 and 1. We limited our analysis to the genes that had measurable expression and appeared in our prior model, and obtained a pseudotrajectory of expression values for 11165 genes across 186 patients. We also created pseudotrajectories for *n*_*g*_ = 500, 2000, and 4000 genes by subsetting to the *n*_*g*_ highest variance genes.

#### 3.2 Model setup for training and testing

We noted that the data set contained 185 different transition pairs, where a transition pair consists of two consecutive expression vectors in the data set $\left(g\left(t_{i}\right),g\left(t_{i+1}\right)\right)$. We split up the 185 transition pairs into training (170, 90%), validation (8, 5%), and test (7, 5%); Please find further implementation details in Supp. Methods 1 and our GitHub repository [34].

For prior domain knowledge model we used the simple linear model: ${𝒫}^{*}\left(\gamma_{k}\right)=W_{0}\cdot \gamma_{k}-\gamma_{k}$. We based our choice of $W_{0}$ on a motif map, similar to that used in the breast cancer analysis in OTTER [61]. The network $W_{0}$ is derived from the human reference genome, for the breast tissue specifically. $W_{0}$ is a binary matrix with $W_{0_{i,j}}\in \{0,1\}$ where 1 indicates a TF sequence motif in the promoter of the target gene. Sequence motif mapping was performed using the FIMO software [62] from the MEME suite [63] and the R package GenomicRanges [64]. Note that $W_{0}$ carries no sign information so that we cannot infer whether TFs inhibit or activate the expression of a gene. Hence we assigned each of the non-zero entries as +1 or −1 with uniform probability.

Validation of explainability was challenging, since there are only few data sets that have ChIP-seq data for many TFs from the same cells. We used ChIP-seq data from the MCF7 cell line (breast cancer, 62 TFs) in the ReMap2018 database [39] to create a validation network of TF-target interactions. We calculated AUC values by comparing the encoded GRNs retrieved from the trained models (see Supp. Methods 2) to the validation network.

#### 3.3 Gene influence scores

Given ${ℳ}_{n_{g}}$ a PHOENIX model trained on the pseudotrajectory consisting of only the $n_{g}$ most variable genes $\left(n_{g}\in \{500,\;2000,\;4000,\;11165\}\right)$, we performed perturbation analyses to compute gene influence scores $ℐ{𝒮}_{n_{g},j}$. We randomly generated 200 initial (i.e. t=0) expression vectors via i.i.d standard uniform sampling ${\left\{g(0{)}_{k}\in R^{n_{g}}\right\}}_{k=1}^{200}$. Next, for each gene j in ${ℳ}_{n_{g}}$, we created a perturbed version of these initial value vectors ${\left\{g^{j}(0{)}_{k}\right\}}_{k=1}^{200}$, where only gene j was perturbed in each unperturbed vector of ${\left\{g(0{)}_{k}\right\}}_{k=1}^{200}$. We then fed both sets of initial values into ${ℳ}_{n_{g}}$ to obtain two sets of predicted trajectories ${\left\{{\left\{\hat{g}(t{)}_{k}\right\}}_{t\in T}\in R^{n_{g}}\right\}}_{k=1}^{200}$ and ${\left\{{\left\{{\hat{g}}^{j}(t{)}_{k}\right\}}_{t\in T}\in R^{n_{g}}\right\}}_{k=1}^{200}$ across a set of time points T. We calculated influence as the average absolute difference between the two sets of predictions, which represented how changes in initial (t=0) expression of gene j affected subsequent (t>0) predicted expression of all other genes in the $n_{g}$-dimensional system:

$$ℐ{𝒮}_{n_{g},j}=\frac{1}{200}\sum_{k=1}^{200}\;\left[\frac{1}{\left|T\right|}\sum_{\begin{array}{c} t\in T\\ t\neq 0 \end{array}}\;\left(\frac{1}{n_{g}}\sum_{\begin{array}{c} i=1\\ i\neq j \end{array}}^{n_{g}}\;\left|{\hat{g}}_{i}(t{)}_{k}-{\hat{g}}_{i}^{j}(t{)}_{k}\right|\right)\right]$$

#### 3.4 Pathway influence scores

Having computed gene influence scores $ℐ{𝒮}_{n_{g},j}$ for each gene j in each dynamical system of dimension $n_{g}$ genes, we translated these gene influence scores into pathway influence scores. We used the Reactome pathway data set, GO biological process terms, and GO molecular function terms from MSigDB [65], that map each biological pathway/process, to the genes that are involved in it. For each system of size $n_{g}$, we obtained the pathway (p) influence scores $\left(𝒫{𝒮}_{n_{g},p}\right)$ as the sum of the influence scores of all genes involved in that pathway:

$${𝒫𝒮}_{n_{g},p}=\sum_{j\in p}\;{ℐ𝒮}_{n_{g},j}$$

We statistically tested whether each pathway influence score is higher than expected by chance using empirical null distributions. We randomly permuted the gene influence scores across the genes to recompute “null” values ${𝒫𝒮}_{n_{g},p}^{0}$. For each pathway, we performed K=1000 permutations to obtain a null distribution ${\left\{𝒫{𝒮}_{n_{g},p,k}^{0}\right\}}_{k=1}^{K}$ that can be compared to $𝒫{𝒮}_{n_{g},p}$. We could then compute an empirical *p*-value as $p=\frac{1}{K}\;\sum_{k=1}^{K}\;I_{𝒫{𝒮}_{n_{g},p,k}^{0}>𝒫{𝒮}_{n_{g},p}}$, where I is the indicator function. Finally, we used the mean $\left(\mu_{0\left(n_{g},p\right)}\right)$ and variance $\left(\sigma_{0\left(n_{g},p\right)}^{2}\right)$ of the null distribution ${\left\{𝒫{𝒮}_{n_{g},p,k}^{0}\right\}}_{k=1}^{K}$ to obtain and visualize pathway z-scores that are comparable across pathways and subset sizes $\left(n_{g}\right)$:

$$z_{\left(n_{g},p\right)}=\frac{𝒫{𝒮}_{n_{g},p}-\mu_{0\left(n_{g},p\right)}}{\sqrt{\sigma_{0\left(n_{g},p\right)}^{2}}}$$

## Figures and Tables

**Table 1:** Qualitative comparison of black-box methods for estimating gene expression dynamics that we benchmarked against PHOENIX *in silico*. We provide details about inputs, learning algorithms, and key performance metrics

			Scalability		Explainability		Notes
	Method	Description of approach for estimating dynamical system	Demonstrated large-scale performance (10^4^+ genes)	Efficient without dimension reduction	Can extract GRN that describes dynamics	Flexibly incorporates prior/domain knowledge	✳ Hyperparameters we optimized using val. set ✂ GRN extraction details

Velocity based (two step)	Dynamo [3]	Uses time-resolved metabolically labelled spliced and unspliced scRNA to estimate RNA velocity (*dx/dt*), then fits a sparse vector field mapping expression to velocity using Gaussian kernel regression	✘ (fewer than 300 genes)	✘ (top30 PC/2D UMAP)	✓	✘	✳ Control points (*M*) and sparsity parameter (λ) ✂ Monte Carlo approach to estimate GRN using Jacobians provided by tool (see Supp. Methods 3.3)

	RNA-ODE [5]	Uses scRNA transcriptome and RNA velocity to fit random forests mapping expression to velocity	✘ (3001 genes)	✓	✓	✘	✳ Number of trees (nTrees) ✂ Tool provides GRN (see Supp. Methods 3.3)

	scDVF [12]	Uses mRNA counts and corresponding and RNA velocity (from scVelo [20]) to fit variational autoencoders mapping expression to velocity	✘ (3000 high var. genes)	✓	✓	✘	✂ Tool provides gene correlation matrix by simulating retrograde trajectories (see Supp. Methods 3.3)

Traj. based (one step)	PRESCIENT [4]	Uses time-series scRNA-seq and cell-growth rate data to learn a potential function Ψ with a neural network. Final drift model is obtained using automatic differentiation *μ* = −∇Ψ	✘ (2500 highly var. genes)	✘ (top50 PC/top30 PC)	✘	✘	✳ Number of neurons inside each hidden layer (*k*_dim_) (see Supp. Methods 3.3)

	Out-of-the-box NeuralODE [6, 17]	Uses time-series expression and NeuralODE out-of-the-box, with traditional activation funcions	✘ (2000 highly expr. genes)	✓	✓	✘	✂ Monte Carlo approach using sensitivity analysis (see Supp. Methods 3.2)

	PHOENIX	Our method	✓	✓	✓	✓	✳ Prior weight (*λ*_prior_) ✂ Algo. described in Supp. Methods 2
